# Whole-genome sequence of *Micrococcus* sp. strain HOU01 isolated from potato root

**DOI:** 10.1128/mra.00268-24

**Published:** 2024-06-25

**Authors:** Van Hong Thi Dao, Loan To Nguyen, Phuong Minh Thi Tran, Truong Xuan Nguyen, Ha Hai Thanh Le, Son Truong Dinh

**Affiliations:** 1 Hanoi Open University, Hanoi, Vietnam; 2 Centre for Animal Science, Queensland Alliance for Agriculture and Food Innovation, The University of Queensland, St Lucia, Brisbane, Queensland, Australia; 3 Faculty of Biotechnology, Vietnam National University of Agriculture, Hanoi, Vietnam; 4 Institute of Agrobiology, Vietnam National University of Agriculture, Hanoi, Vietnam; University of Strathclyde, Glasgow, United Kingdom

**Keywords:** *Micrococcus*, HOU01, endophytic

## Abstract

Endophytes play important roles in potato production. The whole genome of endophytic *Micrococcus* sp. Strain HOU01, isolated from potato root grown at Vietnam National University of Agriculture, Hanoi, Vietnam, was sequenced using Oxford Nanopore’s PromethION platform. The complete circular genome is 2,552,707 bp with a GC content of 72.5%.

## ANNOUNCEMENT

The roots from 15 healthy Solara potato plants were surface sterilized by NaOCl for 20 minutes. 100 µL of the final rinse water was spread on the LB media and incubated at 27°C for 3 days to check whether there were any survived colonies. The surface-sterilized roots were crushed, diluted in liquid LB, and spread on the LB media, and pure culture was accomplished by re-streaking twice on LB agar medium (1).

A single colony of the HOU1 bacteria was grown in liquid LB medium, in a shaker (27°C, 200 rpm) overnight. DNA extraction was performed using a modified protocol in which Phenol:Chloroform:Isoamyl alcohol (25:24:1) was used before adding Chloroform:Isoamyl alcohol (24:1) step (2). The integrity and purity of HOU1 genomic DNA were verified on agarose gel electrophoresis (1% agarose, 150V, 40 minutes). DNA concentration was measured using the Qubit dsDNA Broad Range assay kit (Thermo Fisher Scientific). Genomic DNA was sequenced with Oxford Nanopore’s PromethION sequencer, version R10.4.1 promethION flow cell was used.

The library was prepared following the Native Barcoding Kit 24 V14 protocol (SQK-NBD114.24 kit) from Oxford Nanopore Technologies (Oxford, United Kingdom) with modifications that included extending the incubation period for both end-repair and ligation steps to 30 minutes, instead of the usual 5 minutes. The size-selection step was not performed. To reduce DNA loss due to low input, a 1:1 ratio of DNA to AMPureXP beads (Beckman Coulter, USA) was used. In addition, a short fragment buffer was employed during the ligation wash step. No wash-step was introduced between the end-repair and barcode ligation stages. DNA shearing was minimized by employing bore tips and substituting vortexing steps with gentle flicking of the sample. Basecalling was performed using Guppy v6.4.6 “super accurate” mode, and Chopper version 0.5 (3) was used to remove any reads under 500 bp. 552,777 raw reads were generated and an N_50_ value of the reads was 3,165 base pairs.

The reads were assembled *de novo* using Flye v. 2.9-b1768 (4). The quality and completeness of assembly were evaluated using BUSCO v5.4.5 (5) in prok_genome mode (The lineage data set is bacteria_odb10, creation date: 2020-03-06, number of genomes: 4085, number of BUSCOs: 124) which revealed the HOU01 sequence is >98.4% complete. Using the Type Strain Genome Server (https://tygs.dsmz.de/, version v391), the HOU1 was most matched to the *Micrococcus luteus* NCTC 2665, accession: GCA_006094415 (6).

Using Flye version 2.9, the assembly of the *Micrococcus* sp. strain HOU01 genome revealed a single circular chromosome that spans 2.552.707 bp, with a mean coverage of 171. The genome was annotated to have 2.347 putative genes, as predicted by NCBI Prokaryotic Genome Annotation Pipeline, version 6.6 (7–9).

The strain HOU1 putatively has five biosynthetic gene clusters encoding for the biosynthesis of Terpene, non-alpha poly-amino acids (NAPAA), Betalactone, RRE-containing, and Ectoine, which was predicted by the antiSMASH tool, version 7.1.0 (10). Default parameters were used for all software unless otherwise noted.

## Data Availability

The whole-genome sequence and associated data have been deposited at GenBank (accession number CP141211) and the Sequence Read Archive (SRR26992902).
